# Patients successfully lose body weight after primary total knee arthroplasty but not more than a matched general population

**DOI:** 10.1007/s00402-022-04601-3

**Published:** 2022-09-06

**Authors:** Maria Anna Smolle, Ewald Musser, Georg Hauer, Ines Vielgut, Lukas Leitner, Reinhard Ehall, Andreas Leithner, Patrick Sadoghi

**Affiliations:** 1 Department of Orthopaedics and Trauma, Medical University of Graz, Auenbruggerplatz 5, 8036 Graz, Austria; 2 Department of Orthopaedics, LKH Bad Radkersburg, Dr. Schwaiger-Straße 1, 8490 Bad Radkersburg, Austria

**Keywords:** Obesity, Body mass index, Total knee arthroplasty, Weight loss, Weight trend

## Abstract

**Introduction:**

High BMI is associated with increased risk for knee osteoarthritis, ultimately necessitating total knee arthroplasty (TKA). The aim of this retrospective study was to (1) analyse the amount of postoperative long-term weight loss as reflected by BMI change in TKA patients, (2) identify factors associated with increased change in BMI, and to (3) compare changes with BMI trends of a general population.

**Materials and methods:**

Overall, 298 TKA patients [198 females; mean age: 65.1 ± 7.9 years, median follow-up 8.8 (interquartile range: 5.9–10.8 years)] were included in the final evaluation and compared with an age group-matched control group from the general population regarding weight trends between 2006 and 2014. Main variable of interest in both cohorts was body mass index (BMI). Linear regression analyses were performed to assess changes in weight and BMI over time between TKA patients and the general population. Furthermore, mixed linear-effects models were constructed to analyse the potential change in BMI independent from age and gender.

**Results:**

In TKA patients, a significant drop in BMI by 0.8 ± 3.2 points from postoperative to final follow-up was observed (*p* < 0.001), with reduction being significant independently from age (*p* = 0.382), gender (*p* = 0.310), or revision surgery (*p* = 0.195). In the general population, likewise a significant BMI-decrease by 0.7 ± 6.1 points was observed between 2006 and 2014, with younger people (*p* = 0.004) and females (*p* < 0.001) being more likely to reduce BMI. Yet, BMI-decrease between TKA patients and the general population over time was comparable (*p* = 0.734). Notably, patients with initially higher BMI were significantly more likely to lose weight postoperatively than normal-weight patients (*p* < 0.001).

**Conclusions:**

Our results point against the notion that TKA patients lose a considerable amount of weight in comparison to the general population as soon as improved joint function and pain relief have been achieved. Thus, individualized patient education programmes should be reinforced, promoting a healthy lifestyle.

## Background

Total knee arthroplasty (TKA) constitutes an effective treatment of advanced knee osteoarthritis (OA), Leading to pain relief, maintenance of everyday mobility and regain of independence [12].

In knee OA, reduced mobility and lack of movement causes subsequent joint problems and internal comorbidities, such as cardiovascular disease, diabetes, and obesity [12, 24]. High BMI itself constitutes an independent risk factor of OA, although underlying pathophysiological processes are still largely unknown [4, 5, 9, 12, 27]. It is supposed that excessive joint loading in obese patients alters movement patterns that lead to malalignment and ultimately cartilage damage [5, 25]. In addition, obesity-related dyslipidemia activates pro-inflammatory cytokines and apokines, further causing joint damage [5, 25].

Of note, in the developed world, the proportion of overweight people has steadily increased, as has the amount of TKAs [5]. Maximum therapeutic success with TKA can be achieved by carefully considering important risk factors, such as body weight, osteoporosis, rheumatoid arthritis, neuropathic arthropathy, local osteomyelitis, peripheral vascular diseases, acute and chronic infections prior to surgery [8, 11, 13–16, 26]. Whilst the latter factors cannot be directly targeted, obesity itself may be approached via preventive measures well in advance to maintain patients’ activity and mobility levels. Preoperative weight loss by diet modification alone is often insufficient in advanced stages of knee OA, considering that physical activity is limited by OA-associated pain. Therefore, knee OA-patients who are willing to lose weight may actually do so after TKA, especially after having been counselled toward the increased perioperative risk associated with high BMI.

The purpose of this study was to analyse whether BMI of patients with knee OA changes after TKA. It was hypothesized that patients who had received TKA will have a larger decease in BMI at last follow-up in comparison to BMI-trends observed in the general population during the same time period.

## Methods

**Fig. 1:**
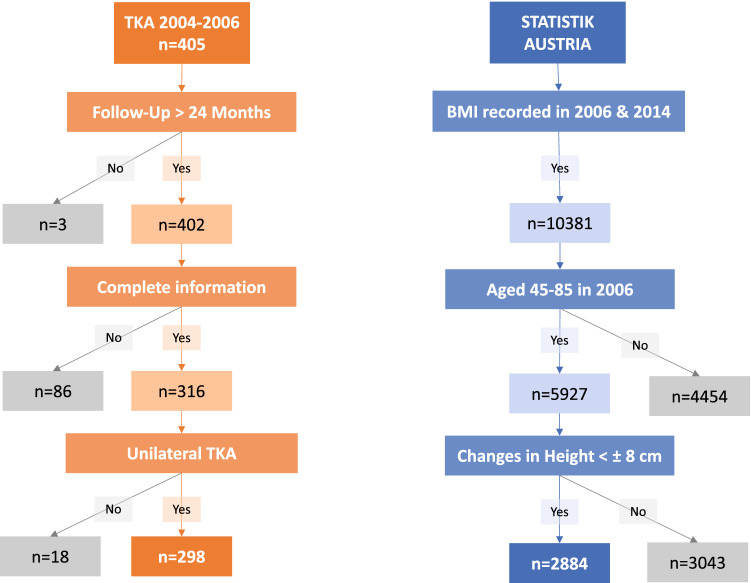
Flow chart for patient selection. Flow-chart showing selection of TKA patients (left) and Statistik Austria cohort (right)

In this analysis, all patients from one orthopaedic department who had received the uncemented Advanced Coated System (ACS®; Implantcast GmbH, Buxtehude, Germany) TKA from 2004 to 2006, were retrospectively assessed (*n* = 405). Three-hundred-sixteen patients were further analysed, after excluding patients with a follow-up of less than 24 months (*n* = 3) and those with incomplete data (e.g., patients with an excessive or implausible alteration in body size (± 8 cm), as documented in records; *n* = 86). Postoperative weight records of those patients who had undergone bilateral TKA were included in the calculations after the second TKA, whilst the first TKA was not considered (*n* = 18). This resulted in a group of 298 patients with TKA for the final analysis (Fig. 1). Median follow-up was 8.8 years [interquartile range (IQR): 5.9–10.8 years], 198 patients were female (66.4%), and the mean age of patients at time of surgery was 65.1 ± 7.9 years (Table 1).

**Table 1:** Demographic variables of patients included in the analysis (*n* = 298)

	Count (%)
Age at surgery (in years; mean ± SD)	65.1 ± 7.9
Gender	
Female	198 (66.4)
Male	100 (33.6)
Side	
Left	176 (59.1)
Right	122 (40.9)
Size femoral component	
2	8 (2.7)
3	123 (41.4)
4	83 (27.9)
5	64 (21.6)
6	19 (6.4)
Size tibial component	
3	30 (10.1)
4	133 (44.8)
5	75 (25.2)
6	59 (19.9)
Preoperative weight (in kg; mean ± SD)	86.5 ± 15.6
Preoperative BMI (in points; mean ± SD)	31.1 ± 4.8
Follow-up (in years; median, IQR)	8.8 [5.9–10.8]
Weight latest follow-up (in kg; mean ± SD)	83.8 ± 17.1
BMI latest follow-up (in points; mean ± SD)	30.3 ± 5.3
Revision surgery	
No	264 (89.2)
Yes	32 (10.8)

The weight trend, represented by the BMI, of the general population in Austria from 2006 to 2014 was compared with the one of TKA patients. Body weight, size and resulting BMI were documented in our patients during clinical follow-up appointments.

The collective of Statistik Austria (Bundesanstalt Statistik Österreich, Vienna, Austria), a non-profit governmental organisation providing—amongst other information—health survey data of people living in Austria obtained on a sample basis in 2006 and 2014, initially comprised a total number of 10,381 people aged between 35 and 85 years in whom health-related data was available at both time points. With people recruited based on supply regions, the cohort is considered representative of the Austrian population.

After excluding those people younger than 45 or older than 85 years in 2006 (all TKA-patients in our study were aged between 45 and 85 years; *n* = 4454), and those with implausible changes in body size (*n* = 3043), 2884 people could be included in the final, age group matched cohort. Upon statistical analysis, both TKA and Statisitk Austria cohort were subdivided into a younger (< 65 years) and older age group (≥ 65 years).

The general weight classes “underweight” (BMI < 18.5), “normal-weight” (BMI 18.5–24.9), “pre-overweight” (BMI 25.0–29.9), “overweight class I” (BMI 30.0–34.9), “overweight class II” (BMI 35–39.9) and “overweight class III” (BMI > 40) were used as defined by the WHO (World Health Organization), with obesity classified as a BMI ≥ 30 [12, 22, 29]. As previously reported [6], a change in BMI from ± 5% of the initial BMI was considered as clinically relevant.

All methods were carried out in accordance with relevant guidelines and regulations. The current study has been approved by the local institutional review board (IRB number: 26–527 ex 13/14). Informed consent had been obtained from all patients prior to study participation.

### Statistical analysis

Statistical analyses were performed with Stata Version 13.0 (StataCorp, TX, US), using paired *t* tests to assess the general trend of weight loss for patients with TKA pre- and postoperatively, as well as for people from Statistik Austria between 2006 and 2014. Means were reported with corresponding standard deviations and medians with IQRs. Paired t tests were performed to assess significant changes in body weight and BMI over time. Linear regression analysis was performed to assess differences between TKA patients and people from Statistik Austria regarding changes in weight and BMI over time, as well as between initial BMI categories and BMI changes during follow-up. Furthermore, mixed linear regression models with random effects were constructed to assess the independent effect of demographic variables (age, gender) on change in BMI (i.e., from preoperative to last follow-up for the TKA cohort, and from 2006 to 2014 for the Statisitk Austria cohort) over time. For the former cohort, the potential impact of revision surgery upon follow-up on change in BMI was likewise assessed in the univariate analysis. A *p* value of < 0.05 was considered statistically significant.

## Results

**Fig. 2:**
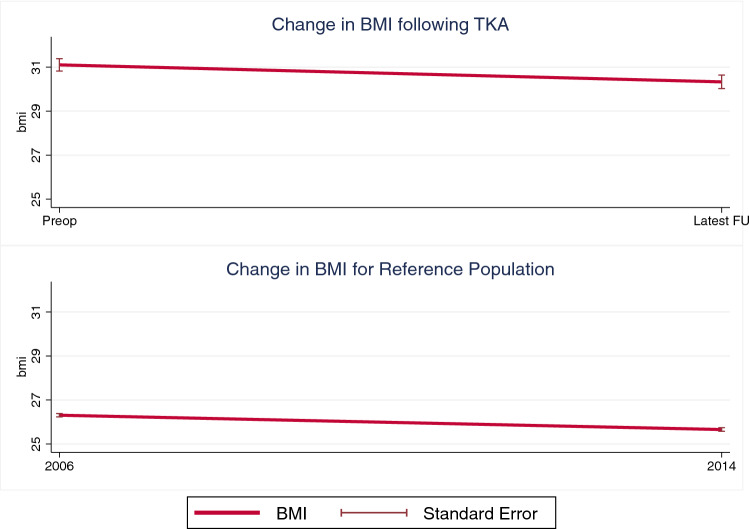
BMI change over time. Overall trend in BMI change over time for TKA patients (top) and reference population from Statistik Austria (bottom)

In the TKA group, 133 patients had been younger than 65 years (44.6%) at time of TKA, and 165 older than 65 years (55.4%). In 32 patients, a revision surgery became necessary during follow-up (10.8%; Table 1). Overall, mean preoperative weight of 86.5 ± 15.6 kg was significantly higher than at last follow-up 83.8 ± 17.1 kg with a mean weight loss of 2.8 ± 9.1 kg (*p* < 0.001). Mean preoperative BMI was 31.1 ± 4.8, and mean BMI at last follow-up 30.3 ± 5.3, with a statistically significant decrease of 0.8 ± 3.2 BMI points (*p* < 0.001; Fig. 2). In other words, 112 (37.6%) TKA patients presented with a decrease in BMI of > 5% in comparison to the preoperative BMI, 122 (40.9%) patients showed alterations in BMI less than 5% of the original BMI, and 64 (21.5%) TKA patients experienced an increase in BMI > 5%. Notably, between males and females there was no difference in gain (23.0% vs. 20.7%), maintenance (46.0% vs. 38.4%) or loss of BMI (31.0% vs. 40.9%) during follow-up (*p* = 0.242).

In the Statistik Austria cohort, 1576 patients were female (54.6%) and 1308 male (45.4%). In 2006, 1702 patients had been younger than 65 years (59.0%) and 1182 older than 65 years (41.0%). Furthermore, in 2006, mean body weight was 75.2 ± 13.6 kg, as compared to a mean body weight of 73.7 ± 14.1 kg in 2014, resulting in a statistically significant decrease of 1.6 ± 17.9 kg (*p* < 0.001). Mean BMI in 2006 and 2014 was 26.3 ± 4.2 and 25.7 ± 4.4, respectively, resulting in a significant decrease of 0.7 ± 6.1 BMI points (*p* < 0.001; Fig. 2). This amounted to 1350 (46.8%) people with a decrease in BMI of > 5% from 2006 to 2014, 503 people with no relevant change in BMI (17.4%), and 1031 people with increase of > 5% (35.8%). Interestingly, males in the Statistik Austria cohort were less likely to gain BMI over time in comparison to females (30.5% vs. 40.1%), whilst being more likely to maintain (19.5% vs. 15.7%) or even lose BMI (50.0% vs. 44.2%).

Between the TKA cohort and people from Statistik Austria, there was no significant overall difference in terms of change in weight (−2.8 ± 9.1 vs. −1.6 ± 17.9; *p* = 0.269) or BMI (−0.8 BMI points vs. −0.7 points; *p* = 0.734) over time.

### Change in BMI depending on preoperative BMI

**Fig. 3:**
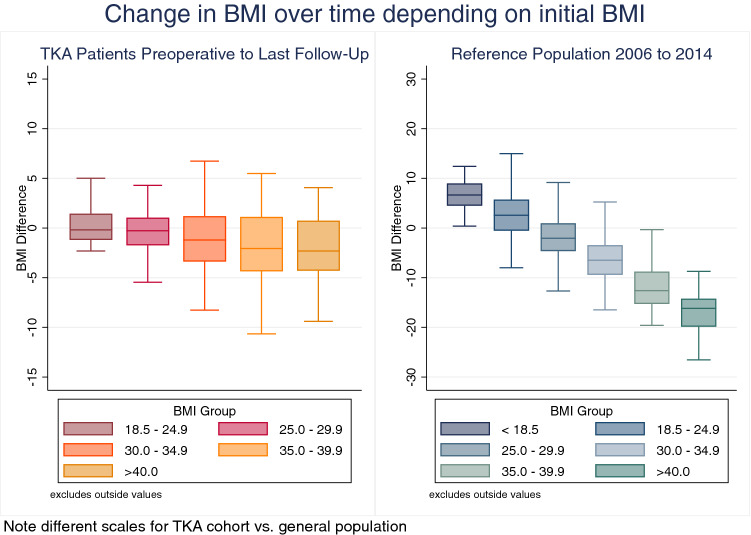
Association between initial BMI and BMI reduction over time. The higher the initial BMI categories—defined according to the WHO—the more pronounced was the BMI reduction at last follow-up for TKA patients (left) and in 2014 for the Statistik Austria cohort (right)

According to linear regression analysis, there was a significant negative association in the TKA cohort for BMI change over time depending on preoperative BMI category (*F*(1296) = −0.708; *p* < 0.001), i.e., the higher the preoperative BMI, the more likely patients presented with reduced BMI at final follow-up (Fig. 3).

In the Statistik Austria cohort, likewise a significant negative association between initial BMI category and change in BMI from 2006 to 2014 was present (*F*(1,2882) = −4.721; *p* < 0.001; Fig. 3), again indicating that people with initial high BMI are more likely to lose weight with time.

### Change in BMI depending on patient age, gender or revision surgery

To assess any time-dependent changes in weight and BMI between males and females, as well as younger and older patients, and depending on revision surgery during follow-up, linear mixed models were constructed.

In the TKA cohort, there was no significant difference in BMI change over time depending on patient age at surgery ± 65 years (*p* = 0.403). Neither there was a difference between males and females regarding change in BMI over time (*p* = 0.327), or whether they had undergone revision surgery during follow-up (*p* = 0.195).

Including age and gender as independent variables in the multivariate linear mixed model, there was a significant BMI reduction of 0.7 points from preoperative to last follow-up for patients with TKA (*p* = 0.001), independent from patient age (*p* = 0.382), or gender (*p* = 0.310; Table 2).

**Table 2:** Multivariate linear effects model showing change of BMI from preoperative to last follow-up for patients with TKA (*n* = 298)

BMI	Coefficient	SE	95% Conf. interval	*P* value
Time				
Preoperatively (Ref.)				**0.001**
Latest follow-up	−0.69	0.21	[−1.10; −0.27]	
Age				
< 65 years (Ref.)				0.382
≥ 65 years	−0.31	0.35	[−1.00; 0.38]	
Gender				
Male (Ref.)				0.310
Female	0.59	0.58	[−0.55; 1.73]	
Constant	31.29	1.12	[29.10; 33.47]	< **0.001**

Significant *p*-values highlighted in bold

*BMI* body mass index, *SE* standard error

In the Statistik Austria group, a BMI increase in 2014 was significantly more often present in patients older than 65 years in 2006 (+ 0.4 BMI points; *p* = 0.001). On the other hand, female patients were more likely to have a lower BMI in 2014 in comparison to 2006 (−1.2 BMI points; *p* < 0.001). In the multivariate linear mixed model, a significant reduction in BMI by 0.5 points from 2006 to 2014 was observed (*p* < 0.001), with both patient age < 65 years in 2006 (*p* = 0.004) and female gender (*p* < 0.001) remaining significant factors associated with BMI reduction (Table 3).

**Table 3:** Multivariate linear mixed effects model on change of BMI from preoperative to last follow-up for people from Statistik Austria (n = 2884)

BMI	Coefficient	(SE)	95% Conf. interval	*P* value
Time				
2006 (ref.)				< 0.001
2014	−0.45	0.11	[−0.69; −0.24]	
Age				
< 65 years (ref.)				0.004
≥ 65 years	0.36	0.12	[0.11; 0.60]	
Gender				
Male (ref.)				< 0.001
Female	−1.16	0.12	[−1.38; −0.93]	
Constant	28.42	0.26	[27.9; 28.9]	< 0.001

*BMI* body mass index, *SE* standard error

## Discussion

This study aimed at analysing whether body weight of patients with knee OA, reflected by BMI, changes after TKA. It was hypothesized that patients can lose a sufficient amount of body weight after TKA surgery. We found that patients significantly lose weight following TKA, as reflected by a mean reduction of 0.8 BMI points, without a significant influence of gender or age. However, the reduction in BMI is comparable to the mean change in BMI of −0.7 points observed in a general population during a similar time frame.

According to previous studies on TKA patients, a BMI reduction exceeding 5% of the preoperative BMI 2 years after TKA was observed in 14% [6] to 21% [28] of patients only, as compared with 37.6% in the current study. However, similar to other authors [3, 21], we likewise discovered that initially obese patients were more likely to lose weight in comparison to TKA patients with normal- to pre-obese BMI [6, 36]. Nevertheless, still 62.4% of patients in our study did not lose or did even gain weight following TKA, a finding also reported by Razzaki et al. [23]. This observation may raise concerns, as obese patients are in general at higher risk for complications following TKA including periprosthetic joint infection, superficial wound healing deficit, venous thrombosis, and injuries of ligamentous structures in comparison to normal-weight patients [3–5, 7, 12, 20, 26, 30, 32, 35]. Furthermore, overweight patients are at increased risk for revision surgery following TKA, with a revision rate of 7% in comparison to 2% for normal-weight patients [4, 5, 10]. Of note, the overall revision rate in our study was 10.8% at 8.8 years, being at the upper limit of previously reported ones [2, 5]. In a study on morbidly obese TKA patients with a BMI > 50, postoperative complications were threefold higher than in normally weight TKA patients [21]. In addition, morbidly obese TKA patients had a ninefold increased risk for aseptic loosening as compared with non-obese patients, albeit these results did not reach statistical significance [21].

Satisfaction of TKA patients is not only influenced by potential surgery-associated complications, but also the postoperative outcome as reflected by functional and quality of life scores. Whilst the significant correlation between increased complication risk and high BMI is evident, the influence of obesity on postoperative functional and quality of life (QoL) outcome scores in TKA patients is less pronounced. In detail, overweight class I–III patients tend to have similar functional and QoL outcome scores [5, 6, 19], whereas functionality significantly worsens in morbidly obese in comparison to normal-weight TKA patients [21].

According to Lachiewicz et al. and Zan et al. older TKA patients are more likely to lose weight following surgery than younger patients [17, 36]. In the current study, however, patient age had no significant impact on postoperative change in BMI. In addition, there was no significant difference between male and female patients with regard to BMI changes. As a comparison, younger people and women in the general population were more likely to present with lower BMI in 2014 in comparison to 2006, which may reflect previously reported gender-specific differences in health literacy and willingness to lose weight [31].

A mean decrease of 0.8 BMI points was observed for the entire TKA cohort following surgery. In other words, 37.6% of patients lost more than 5% of their initial BMI, with this threshold previously defined as a satisfactory criterion for clinically meaningful weight loss [33]. On the other hand, 21.5% of patients gained even more than 5% of preoperative BMI after surgery, albeit this number is lower than observed for the general population (35.8%). Furthermore, whilst the clinical significance of 5% weight loss also depends on parameters as amount of initial weight loss and risk factors to be addressed [1, 33, 34], the current study highlights that just over one third of TKA patients achieve a weight reduction of potential health benefit.

There are limitations to the current study. Owing to its retrospective design, information on change in height, size and resulting BMI was obtained from medical records, anaesthesia protocols and fever charts, whereas measurement errors caused by non-standardised assessment cannot be ruled out, despite excluding those patients with an extreme change in body height over time. Furthermore, as purely focusing on changes in BMI relative to a comparable general population, we did not analyse a potential correlation between BMI change and clinical or functional outcome in our TKA cohort, or other health-related variables and activity levels in comparison to the Statistik Austria cohort, except for the demographic variables age and gender. In addition—apart from revision surgery during follow-up—other potential confounding factors that may alter change in BMI as presence of further degenerative joints, diabetes, hypertension, or depression were not evaluated in this study. Thus, it cannot be ruled out that these unknown confounders may have had an influence on the amount of weight loss following TKA. On the other hand, the course of BMI change can be considered natural, given the study’s retrospective design with no initial specific focus on body weight. In addition, during clinical follow-up, no intermittent weight or BMI data of patients was available, wherefore we rather focused on the long-term weight changes than short-term effects that may be lost with time. Furthermore, it has to be noted that the Statistik Austria cohort—as it is representative of the Austrian population—also potentially included some patients with TKA. Yet, with an incidence of 184 per 100.000 inhabitants per year in 2009, and an incidence of 202 per 100.000 inhabitants per year in 2015, the number of people with TKA in the Statistik Austria cohort may be considered negligible [18].

Nevertheless, strengths of this study can be seen in its single-centre cohort with a median follow-up of 8.8 years, as well as the direct comparison to weight trends in a general population of the same country obtained during a similar time period.

Via this approach we were able to show that albeit a significant drop in BMI has occurred in TKA patients at a median follow-up of 8.8 years, its amount is comparable to the one observed in the general population during a similar time period. Thus, our observations should promote setup of individualised perioperative programmes, encouraging patients toward a healthier lifestyle, thus eventually improving the overall outcome [3, 12, 21].

## Conclusions

At a median of 8.8 years following surgery, TKA patients have lost weight, reflected by a mean decrease of 0.8 BMI points. Yet, this is almost similar to the 0.7 BMI point reduction observed in a comparable general population with weight and BMI trends collected over an 8-year period. Notably, similar to trends in the general population, the higher the preoperative BMI, the more likely TKA patients will lose weight postoperatively. Yet, the current results point against the notion that TKA patients will lose a considerable amount of weight following surgery. Therefore, an individualized patient education programme should be initiated perioperatively in TKA patients to promote a healthy lifestyle postoperatively.

## Abbreviations

TKA: Total knee arthroplasty
BMI: Body mass index
OA: Osteoarthritis
ACS: Advanced Coated System®
IQR: Interquartile range
WHO: World health organisation
